# Progress on Noble Metal-Based Catalysts Dedicated to the Selective Catalytic Ammonia Oxidation into Nitrogen and Water Vapor (NH_3_-SCO)

**DOI:** 10.3390/molecules26216461

**Published:** 2021-10-26

**Authors:** Magdalena Jabłońska

**Affiliations:** Institute of Chemical Technology, Universität Leipzig, Linnéstr. 3, 04103 Leipzig, Germany; magdalena.jablonska@uni-leipzig.de

**Keywords:** noble metals, supported oxides, bifunctional catalysts, NH_3_-SCO

## Abstract

A recent development for selective ammonia oxidation into nitrogen and water vapor (NH_3_-SCO) over noble metal-based catalysts is covered in the mini-review. As ammonia (NH_3_) can harm human health and the environment, it led to stringent regulations by environmental agencies around the world. With the enforcement of the Euro VI emission standards, in which a limitation for NH_3_ emissions is proposed, NH_3_ emissions are becoming more and more of a concern. Noble metal-based catalysts (i.e., in the metallic form, noble metals supported on metal oxides or ion-exchanged zeolites, etc.) were rapidly found to possess high catalytic activity for NH_3_ oxidation at low temperatures. Thus, a comprehensive discussion of property-activity correlations of the noble-based catalysts, including Pt-, Pd-, Ag- and Au-, Ru-based catalysts is given. Furthermore, due to the relatively narrow operating temperature window of full NH_3_ conversion, high selectivity to N_2_O and NO_*x*_ as well as high costs of noble metal-based catalysts, recent developments are aimed at combining the advantages of noble metals and transition metals. Thus, also a brief overview is provided about the design of the bifunctional catalysts (i.e., as dual-layer catalysts, mixed form (mechanical mixture), hybrid catalysts having dual-layer and mixed catalysts, core-shell structure, etc.). Finally, the general conclusions together with a discussion of promising research directions are provided.

## 1. Introduction

Ammonia (NH_3_) is a corrosive, highly toxic, and reactive inorganic gas with a pungent odor under ambient conditions. It is an atmospheric pollutant that is dangerous to health and life because it could corrode skin, eyes, and lungs, and cause permanent injury or even death when the concentration is higher than 300 ppm [1,2]. Ammonia is also reported to be the most common pollutant found in water streams, further affecting human health as the consequence of eating toxic fish and drinking water [3]. The toxic action of ammonia on aquatic animals can lead to the extinction of the entire population, threatening many important ecosystems and fisheries worldwide [4,5]. NH_3_ is referred to as one of four major atmospheric pollutants together with NO*_x_*, SO*_x_*, and nonmethane volatile organic compounds (NMVOC). Approximately 5600 kt y^−1^ of ammonia are emitted into the atmosphere each year, i.e., up to 4-times higher emission levels than in the previous century, and it continues to increase [6]. NH_3_ is emitted by several various processes, including nitric acid production, urea manufacturing, nitrogen fertilizer production, biomass, and coal gasification, petroleum refining and refrigeration, livestock waste, and animal agriculture, transport (as a gas slip from the process of selective catalytic reduction of NO*_x_* using NH_3_ or urea (SCR) in DeNO*_x_* applications), etc. More attention was given to the removal of NH_3_ from gaseous and waste streams, e.g., through its oxidation.

The selective catalytic oxidation of ammonia (NH_3_-SCO) into nitrogen and water vapor is considered as the most promising method for the elimination of NH_3_ from oxygen-containing exhaust gases (Equation (1)) [7]:

4 NH_3_ + 3 O_2_ → 2 N_2_ + 6 H_2_O (1)

NH_3_ is generated by an onboard aqueous urea dosing system. The obtained ammonia acts as a NO*_x_* reductant in the DeNO*_x_* process. There is a serious risk that unreacted ammonia is released into the atmosphere. Thus, the active SCO catalysts (so-called *guard catalyst*, ammonia slip catalyst—ASC, ammonia oxidation catalyst—AMOX) should operate in a broad temperature range (up to 600–700 °C—in the cycle of diesel particulate filter regeneration) in the presence of typical components of the exhausts (H_2_O, CO*_x_*, and SO*_x_*), and additionally should selectively direct the reaction to the formation of N_2_ and H_2_O. Euro VI emission standards for heavy-duty vehicles (HDVs) introduced for the first time limits for NH_3_ emissions up to 10 ppm [8]. Currently, there are no limits for NH_3_ emitted from light-duty vehicles (LDVs, i.e., passenger cars), despite their high levels of emissions (e.g., [9,10]). Thus, potentially NH_3_ will be considered next to ultra-fine particles smaller than 23 nanometers (PN_10_) and nitrous oxide (N_2_O) in the upcoming regulations, e.g., upcoming Euro emission standards.

Various kinds of catalysts were studied for NH_3_-SCO, including noble metals (e.g., Ru [11]), supported noble metals (e.g., Au/Nb_2_O_5_, Au/ZrO_2_ [12]), (mixed) metal oxides, supported metal oxides, modified zeolites (e.g., Pt-CuMgAlO*_x_* [13], Ag-USY [13]), etc. These groups of catalysts investigated in NH_3_-SCO were summarized by Jabłońska et al. [7,14], Gao et al. [15] and Lan et al. [16]. In general, noble metal-based catalysts tend to possess high activity at low temperatures (< 300 °C), while their high cost and relatively low N_2_ selectivity have restrained their widespread application. Transition metal-containing oxides and zeolites show improved selectivity toward N_2_ than noble metal-based catalysts; however, they need higher operating temperatures (300–600 °C). Consequently, the proper combination of these two metals (in the form of bifunctional catalysts) could produce the catalysts with enhanced activity and N_2_ selectivity. In general, the concept of bifunctional catalysts is based on the internal selective catalytic reduction (i-SCR) mechanism. This mechanism consists of two main steps. In the first step, part of ammonia is oxidized to NO and NO_2_—minor by-product, while in the second step, NO and NO_2_ are reduced by ammonia (unreacted in the first step) with the formation of N_2_ and H_2_O (DeNO*_x_*, NH_3_-SCR). In this step, also the formation of N_2_O is possible. Besides the i-SCR mechanism, other major reaction pathways, i.e., the imide (NH, in which NH_3_ transforms to N_2_ and N_2_O as final products, with nitrosyl (HNO) as an intermediate) mechanism and the hydrazine (with the formation of a hydrazine-type (N_2_H_4_) intermediate) mechanism were proposed for NH_3_-SCO over different types of catalysts. The details of the above-mentioned reaction mechanisms can be found in previous review articles (e.g., [7,14]). Additionally, due to lack of identification of the characteristic intermediates of the aforementioned three mechanisms, recently Wang et al. [17] proposed a N_2_^−^ mechanism of NH_3_-SCO on a Ag/nano-Al_2_O_3_. Li et al. [18] postulated an Eley–Ridel mechanism over perovskite-based catalysts, where gaseous NH_3_ reacts with adsorbed -ONH_2_ species to form the surface diazo species (-N=N-) with the rate-determining step depending on the catalysts composition.

Recent interest focuses on bifunctional catalysts consisting of noble metal-based catalyst and transition metal-based catalyst. Previous review articles, including ammonia oxidation, give a clear statement about high activity, N_2_ selectivity, and stability over Cu-containing materials (Jabłońska et al., 2016, Jabłońska, 2020) [7,19]. From the noble metal-based catalysts the most frequently applied—Pt/Al_2_O_3_, provides high activity but also significant selectivity to byproducts (N_2_O and NO*_x_*). Besides Pt/Al_2_O_3_, also the Ag/Al_2_O_3_ catalyst has received extensive concerns on the low-temperature NH_3_-SCO. Other systems were less investigated for NH_3_-SCO than Pt/Al_2_O_3_ or Ag/Al_2_O_3_. Thus, the present mini-review aims to provide a broad picture of the property-activity correlations of noble metal-based catalysts investigated for NH_3_-SCO up to now (including Pt-, Pd-, Ag- and Au-, Ru-based catalysts). The NH_3_-SCO catalysts are classified considering their full NH_3_ conversion and N_2_ selectivity in the same temperature range. If not provided in the references, the catalytic activity and N_2_ selectivity were roughly estimated based on NH_3_ conversion-temperature profiles. This overview will shed light on future research directions regarding catalyst composition and architecture that maximizes the oxidation of NH_3_ into N_2_ over a broad temperature range and in the presence of the typical components of exhaust, such as H_2_O, SO*_x_*, and CO*_x_*.

## 2. Pt- and Pd-Based Catalysts

**Figure 1:**
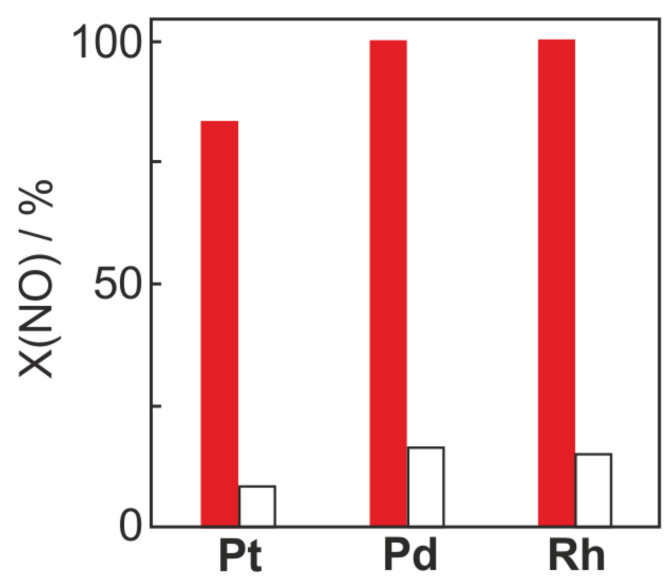
Conversion of NO over the PGMs on pulsing of *c*(^15^NH_3_):*c*(NO):*c*(Ne) = 1:0.2:1 (solid bars) and *c*(^15^NH_3_):*c*(NO):*c*(Ne) = 1:2:1 (open bars) at 800 °C. Reprinted from [25] with permission from Elsevier.

Early work on NH_3_ oxidation was given by Il’chenko et al. [20] Among metal-based catalysts, Pt and Pd are the most active for the ammonia oxidation (p(NH_3_) = 0.1 atm, p(O_2_) = 0.9 atm) but also the most selective to N_2_O—according to the following order: Pt, Pd > Ni > Fe > W > Ti (note that Rh was not mentioned in Il’chenko review). The catalytic ammonia oxidation over platinum is a key step in the industrial manufacturing of nitric acid. The ammonia oxidation exhibits a moderate structure sensitivity, while the activity decreased in the order of: Pt foil > Pt(865) > Pt(533) > Pt(443) > Pt(100) due to different oxygen sticking coefficient [21]. Novell–Leruth et al. [22] used periodic slab density functional theory (DFT) calculations and found that NH_3_ adsorbs preferentially on the top sites, NH_2_ (dehydrogenation intermediates) on the bridge sites, while NH and N species on the hollow sites on both the (111) and (100) surfaces. The ammonia oxidation with atomic or molecular oxygen over Pt(100), Pt(111), stepped Pt(111)/Pt(211) or terrace Pt(111) orientations, etc., yields N_2_, NO, N_2_O, and H_2_O, in varying amounts depending on reactant conditions. Steady-state reaction studies [23] under ultra-high vacuum (UHV) conditions with a stepped Pt(111) surface revealed that excess NH_3_ lead to N_2_ formation, while under excess O_2_, NO formation was preferred. No other nitrogen-containing products, i.e., N_2_O were detected in the gas phase (note that N_2_O was never observed under UHV conditions [24]). Similar conclusions were given by Pérez–Ramirez et al. [25], who studied the sequence of steps in NH_3_ oxidation (applying the isotope ^15^NH_3_) over Pt, Pd, and Rh wires in the temporal analysis of products (TAP) reactor at relevant temperatures in industrial ammonia burners. High NO selectivity is favored at a high ratio of adsorbed *n*(O)/*n*(NH*_x_*) species, e.g., at *c*(O_2_)/*c*(^15^NH_3_) = 0.1, the ^15^NO selectivity over Pt reached 45%, while at *c*(O_2_)/*c*(^15^NH_3_) = 10—ca. 100% selectivity. NO was found to be a primary reaction product in NH_3_ oxidation, while N_2_ and N_2_O originate from consecutive NO transformations. Pd and Rh were more active for the reduction of nitric oxide by ammonia than Pt (Figure 1). Additionally, DFT calculations showed that the N_2_O formation over Rh(100) plane needs higher activation energy than over Pt(100) or Pd(100). Furthermore, Rh(100) was more active in NH_3_ decomposition (possessed a lower activation barrier for the NH_3_ → NH_2_ step) than Pt and Pd surfaces, and strongly stabilized the dehydrogenated NH and N species [22].

γ-Al_2_O_3_ and ZSM-5 are often used as the supports for noble metal-based catalysts for the selective catalytic oxidation of NH_3_. A summary of Pt-, Pd-based catalysts is presented in Table 1. Pt/Al_2_O_3_ is usually applied to provide high low-temperature activity. The main drawback of such catalyst is low N_2_ selectivity due to the formation of N_2_O (below 250 °C) and NO*_x_* (above 250 °C). The metallic Pt is significantly more active for NH_3_-SCO than oxidized platinum [26,27], which provide limited sites for O_2_ dissociation [28]. The operando XANES/EXAFS studies revealed the highest N_2_ selectivity (ca. 80%) over H_2_-reduced (2 wt.%)Pt/Al_2_O_3_. Nevertheless, under reaction conditions, at least 40% of Pt surface remains oxidized resulting in the formation of N_2_O [29]. Otherwise, the metal-support interactions of Pt/TiO_2_ were reported to stabilize Pt in the metallic state (also under reaction conditions) [30]. The Pt^0^ content can also be manipulated by preparation procedure of Pt/SiO_2_-Al_2_O_3_, i.e., by adding ascorbic acid (vitamin C, vC; *n*(vC)/*n*(Pt) = 0.25–1.5) [31]. Ostermaier et al. [26] reported that small Pt particles (2.0 and 2.7 nm) of (1–2.93 wt.%)Pt/Al_2_O_3_ demonstrate lower activity in comparison to larger crystallites (15.5 nm). The catalysts with a small size of Pt^0^ crystallites were characterized by the strongest deactivation during NH_3_ oxidation due to their oxidation to PtO*_x_*, where *x* depends on the particle size [32]. Later, Sobczyk et al. [33,34] demonstrated with positron emission profiling (PEP) that the catalysts deactivate due to poisoning of the surface mainly by nitrogen species (NH and NH_2_).

**Figure 2:**
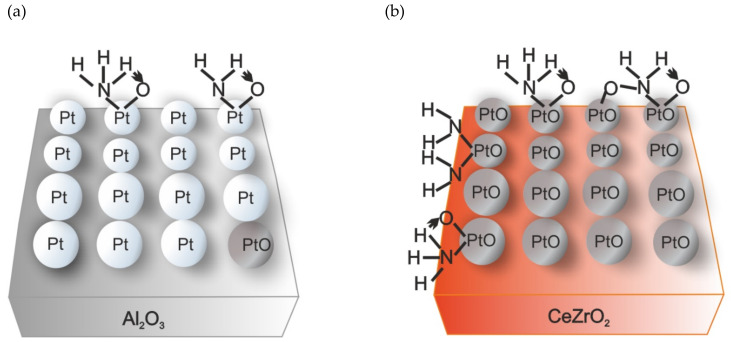
(**a**) Reaction mechanisms and Pt states over Pt/Al_2_O_3_ and (**b**) Pt/CeZrO_2_. Reprinted from [42] with permission from ACS Publications.

The dispersion of Pt species was increased after the introduction of ethylenediamine (from 2.8 to 2.0 nm) during the preparation of (1 wt.%)Pt/SiO_2_-Al_2_O_3_ [35], and thus lead to higher activity in NH_3_-SCO in the presence of CO_2_ and H_2_O, compared to that of unmodified samples. The N_2_ selectivity remained nearly unaffected by the Pt particle size. Contrary to these studies, Slavinskaya et al. [36] found that a larger Pt particle size (ca. ~23 nm compared to ~1 nm) of (2 wt.%)Pt/Al_2_O_3_ enhanced activity. Additional measurements in the presence of CO_2_ and H_2_O did not change the trends of activity and selectivity of Pt/Al_2_O_3_ on the Pt dispersion and Pt state. Similar to the above discussed studies over Pt/SiO_2_-Al_2_O_3_, N_2_ selectivity did not depend on the Pt particle size, while in all cases, N_2_ selectivity was below 70% (>300 °C). Furthermore, authors [27] showed no deactivation of the catalysts, i.e., the oxidation state of platinum in Pt/Al_2_O_3_ did not increase after the catalytic experiments. Also, the hydrothermal aging (in a feed containing O_2_, H_2_O, CO_2_, at 550 °C over 250 h) of a Pt/Al_2_O_3_ washcoated monolith did not influence its activity below 250 °C [37]. Above 300 °C the activity significantly decreased with aging time (0, 122, 253 h) but the product selectivity remains the same. Recently, Machida et al. [38] found that a thin-film catalyst, which was prepared by deposing a nanoscale-thickness Pt(111) overlayer on a 50 μm-thick Fe-Cr-Al metal foil (Pt/SUS) achieved more than 180-fold higher TOF compared with the conventional (0.13 wt.%)Pt/Al_2_O_3_. The thermal stability of Pt/SUS was enhanced by the insertion of the Zr layer between the Pt and SUS foil. For the Pt surfaces, the NH mechanism was mostly proposed by experimental and DFT simulation studies; e.g., over Pt(100) or Pt(111) [39,40,41]. Also, the so-called *NH mechanism* (i.e., NH as the intermediates in the imide mechanism) occurred on Pt/Al_2_O_3_, while the *HNO and N_2_H_4_ mechanism* (i.e., HNO and N_2_H_4_ as the intermediates in the imide and hydrazine mechanism, respectively) coexisted on Pt/CeZrO_2_ (Figure 2) [42].

Li and Armor [43] studied a series of zeolite ZSM-5 ion-exchanged with (4.07 wt.%)Pd, (2.66 wt.%)Rh, and (2.55 wt.%)Pt as catalysts for NH_3_-SCO. Among them, relatively high activity and N_2_ selectivity in the low-temperature range (≤300 °C) were found for the Pd-containing catalysts (i.e., full ammonia conversion with 91% N_2_ selectivity at 300 °C in the presence of 5 vol.% H_2_O). 58–61% of N_2_O selectivity on (2.55 wt.%)Pt-ZSM-5 and 16–25% on (4.01 wt.%)Pd-ZSM-5 was obtained at 250–300 °C. The noble metal-exchanged ZSM-5 materials were less affected by water vapor than the corresponding Al_2_O_3_ supported catalysts. Similar results, i.e., 92% NH_3_ conversion and 73% N_2_ selectivity at 350 °C over (5.51 wt.%)Pd-ZSM-5 in NH_3_-SCO (feed without H_2_O), were also reported by Long and Yang [44]. Furthermore, Jabłońska et al. [45] investigated zeolites HY modified with palladium (0.05–2.5 wt.%). An increase in Pd loading leads to higher catalysts activity together with the drop in N_2_ selectivity. The palladium oxide species (PdO*_x_*) were found to be active sites for ammonia oxidation (based on FT-IR studies). A part of ammonia was stabilized against oxidation over the zeolite framework acid sites (in the form of NH_4_^+^), and lead to enhanced N_2_ selectivity in the higher temperature range. The analysis of the results of temperature-programmed (NH_3_-SCO with various spaces velocity) and spectroscopic studies lead to the conclusion that the ammonia oxidation over Pd-Y followed the i-SCR mechanism. On the other hand, an appearance of hydrazine species (intermediates in the hydrazine mechanism) on the ammonia pre-adsorbed Pd-Y catalyst at 250 °C during FT-IR studies, suggested that the ammonia oxidation is more complicated and followed different parallel routes. Otherwise, Wells et al. [46,47] identified PdN*_x_* under reaction conditions over H_2_-reduced (1.5 wt.%)Pd/Al_2_O_3_ and Pd/Y (*n*(Si)/*n*(Al) = 2.6) as the dominant species during N_2_ formation (based on combined operando spectroscopy and DFT calculations). As stated above, palladium-containing materials appear as promising NH_3_-SCO catalysts, although, their stability (also in the presence of H_2_O, SO*_x,_* and CO*_x_*) needs confirmation.

**Figure 3:**
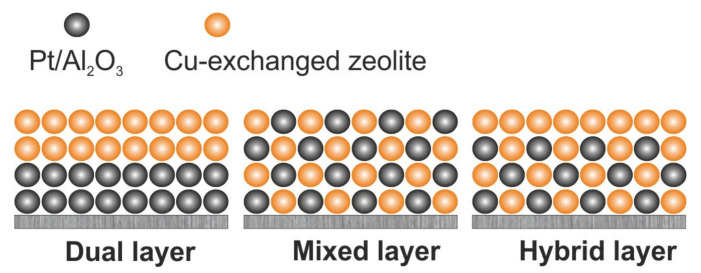
Schematic diagram representing bifunctional ammonia slip catalyst in three different washcoated structured methodologies. Reprinted from [64] with permission from Science Direct.

The state-of-the-art NH_3_-SCO systems include a combination of a noble metal-based catalyst—usually Pt/Al_2_O_3_, and an SCR catalyst, e.g., Cu- or Fe-containing zeolite. Thus, a part of ammonia is oxidized over the noble metal-based catalyst to N_2_ and NO*_x_* which is further transformed to N_2_ over the SCR catalyst. Different arrangements of both metals were reported in the literature, i.e., noble/transition metal deposited on one support in systems such as (0.05 wt.%)Pt/(1 wt.%)Cu/Al_2_O_3_ [48], (0.5–4 wt.%)Pt/(20 wt.%)Cu/Al_2_O_3_ [49], (1 wt.%)Pt/(20 wt.%)Cu/Al_2_O_3_ [50,51,52], (1.5 wt.%)Pt-(5.5 wt.%)Cu/ZSM-5 [53], (0.5 wt.%)Pt-(1.54 wt.%)Fe-ZSM-5 [54], (1.5 wt.%)Pt-(0.5 wt.%)Fe/ZSM-5 [55], (0.21 wt.%)Pt/CuMgAlO*_x_* hydrotalcite-derived mixed metal oxides [13], (2 wt.%)PdO/(5 wt.%)CuO/Al_2_O_3_ [56], etc. Besides such form, the active components may be present in different configurations—dual-layer configuration, mixed and hybrid layer sample types, that are presented in Figure 3. The dual-layer catalytic systems consist of noble metal-based catalyst as a bottom layer, and transition metal-based catalysts as an upper layer, i.e., Pt/Al_2_O_3_ and Cu-ZSM-5, Pt/Al_2_O_3_ and Cu-SSZ-13, Pt/Al_2_O_3_, and Fe-ZSM-5 investigated by Shrestha et al. [57,58,59], Pt/Al_2_O_3_ and Fe-zeolite investigated by Scheuer et al. [60] and Colombo et al. [61]. E.g., Shrestha et al. [57] pointed out the increase of N_2_ selectivity (with a corresponding decrease in NO selectivity) with increasing copper loading of Cu/ZSM-5 (NH_3_-SCR layer), e.g., from 58% to 82% at 250 °C for 0.8 and 2.5 wt.% of Cu, respectively. Still, N_2_O selectivity reached a maximum of about 40% at 260 °C in all cases. Similar N_2_O selectivity was reported over Pt/Al_2_O_3_ and Fe-ZSM-5 arranged as both dual-layer and mixed catalysts [58]. Also, the NH_3_ oxidation depended on the applied conditions, i.e., space velocity (66,000 *versus* 265,000 h^−1^). At higher space velocity mixed catalyst revealed (ca. 7%) higher NH_3_ conversion, while dual-layer catalyst provided higher N_2_ selectivity (especially above 350 °C). The hybrid catalyst (bottom layer of mixed Cu-SSZ-13 + Pt/Al_2_O_3_, top layer of Cu-SSZ-13) allowed to achieve (ca. 5%) higher NH_3_ conversion than that of the dual-layer catalyst [59]. Furthermore, Dhillon et al. [62] applied sacrificial agents (yeast or polymer) to generate macropores on a (2.90 wt.%)Cu-SSZ-13 top-layer washcoat supported on (1.47 wt.%)Pt/Al_2_O_3_, and thus to enhance NH_3_ conversion (still below 90% up to 500 °C) without impact on N_2_ selectivity. Recently, Gosh et al. [63] reported a Pt/Al_2_O_3_@Cu-ZSM-5 (0.05 wt.% Pt, 2.93 wt.% Cu) core-shell catalyst that allowed full NH_3_ conversion at ca. 300 °C and 100% N_2_ selectivity (up to 275 °C). The NH_3_ conversion was negligibly affected by the variation in the shell thickness (0.5 µm *versus* 1.2 µm), while the ticker shell was beneficial in improving N_2_ selectivity at higher temperatures. The addition of H_2_O (5 vol.%) to the feed had a minor impact on the catalyst activity, while more tests in the presence of CO*_x_* and SO*_x_* are still required.

Concluding this part, as can be seen from the above presented data, a great variety of Pt- and Pd-based catalytic systems were developed and tested for NH_3_-SCO. In particular, (1–2 wt.%)Pt/Al_2_O_3_ was reported as one of the most active catalyst that allowed to obtain full NH_3_ conversion around 200–450 °C with N_2_ selectivity of 15–87% (according to data gathered in Table 1), depending on the applied catalyst preparation procedure as well as pretreatment and reaction conditions. Pt^0^ serves as the active species responsible for high catalytic activity, and thus most of the catalytic systems were prepared by an impregnation method and subsequently reduced in H_2_. Great research efforts aimed at determining the role of Pt dispersion in NH_3_-SCO, but there is no clear consensus about it yet. Platinum (0.05–1.5 wt.%) was also applied as the most active noble metal component in the bifunctional catalysts. Such systems fully oxidized NH_3_ in a broad temperature range of 195–500 °C with 44–100% N_2_ selectivity (according to data gathered in Table 1) depending on their architecture, i.e., Pt deposited on one support with transition metal component, as dual-layer catalysts, mixed form, hybrid catalysts or incorporated in the core-shell structure, etc. Among them the (0.46 wt.%)Pt/Al_2_O_3_-(2.5 wt.%)Cu-ZSM-5 dual-layer catalyst (full NH_3_ conversion at 250–500 °C, 82–100% N_2_ selectivity) and (0.05 wt.%)Pt/Al_2_O_3_@(2.93 wt.%)Cu/ZSM-5 core-shell catalyst (full NH_3_ conversion at 310–500 °C, 91–94% N_2_ selectivity) appear as the most interesting systems for further catalysts optimization. However, the stability of these catalysts and the influence of the potential catalyst pollutants usually present in the exhausts, i.e., H_2_O, SO*_x_* and CO*_x_*, were not fully provided within the scope of the studies. A similar conclusion can be given for other Pt- or Pd-containing catalysts (only a few materials were tested in the presence of H_2_O and CO_2_). Hence, further studies are required to understand structure-activity relationships and reaction mechanisms under application-relevant reaction conditions.

**Table 1:** Comparison of full NH_3_ conversion and N_2_ selectivity in same temperature range over Pt-based catalysts reported in literature.

Catalyst	Catalyst Preparation	Reaction Conditions	NH_3_ Conversion_N_2_ Selectivity/% (Temperature/°C)	Ref.
(3 wt.%)Pt-Rh	wash-coated on Al_2_O_3_, calcination, 400 °C, air	0.08 vol.% NH_3_, 4 vol.% O_2_, He balance, GHSV 92,000 h^−1^	100/62% (400 °C)	[65]
(4.4 wt.%)Pt-Rh	wash-coated on Al_2_O_3_, calcination, 500 °C, air	0.1 vol.% NH_3_, 4 vol.% O_2_, He balance, GHSV 92,000 h^−1^	100/80% (400 °C)	[66]
(1.2 wt.%)Pt/Al_2_O_3_	impregnation, calcination, 600 °C, air; reduction conditions not shown)	0.1 vol.% NH_3_, 10 vol.% O_2_, He balance, 50 mL min^−1^, mass of the catalyst: 0.1 g, WHSV 30,000 mL h^−1^ g^−1^	100/75% (200 °C)	[67]
		1.14 vol.% NH_3_, 8.21 vol.% O_2_, He balance, 74.7 mL min^−1^, mass of the catalyst: 0.2 g, WHSV 22,410 mL h^−1^ g^−1^	100/87% (200 °C)	
(1.73 wt.%)Pt/Al_2_O_3_	impregnation, calcination, 400 °C, air; reduction, 250 °C, H_2_	0.1 vol.% NH_3_, 4 vol.% O_2_, He balance, 500 mL min^−1^, mass of the catalyst: 0.145 g, GHSV 120,000 h^−1^	100/40–60% (200–400 °C)	[27]
(2 wt.%)Pt/Al_2_O_3_	impregnation, calcination, 400 °C, air; oxidation, 400 °C, O_2_/He	0.1 vol.% NH_3_, 4 vol.% O_2_, He balance, 500 mL min^−1^, mass of the catalyst: 0.145 g, GHSV 120,000 h^−1^	100/40–50% (325–400 °C)	[36]
	impregnation, calcination, 400 °C, air; reduction, 350 °C, H_2_; calcination, 400 °C, Ar		100/30–60% (250–400 °C)	
(1 wt.%)Pt/Al_2_O_3_	impregnation, calcination, 550 °C, air	0.02 vol.% NH_3_, 10 vol.% O_2_, N_2_ balance, 2.31 l min^−1^, mass of the catalyst: 0.34 g, WHSV 407,000 mL g^−1^ h^−1^	100/25–49% (250–450 °C)	[68]
(1 wt.%)Pt/CeO_2_-SiO_2_			100/15–42% (225–450 °C)	
(1 wt.%)Pt/SiO_2_-Al_2_O_3_	impregnation, calcination, 550 °C, air; monolithic catalyst	0.02 vol.% NH_3_, 10 vol.% O_2_, 8 vol.% CO_2_, 5 vol.% H_2_O, N_2_ balance, GHSV 100,000 h^−1^	100/10–40% (300–450 °C)	[35]
(1 wt.%)Pt/SiO_2_-Al_2_O_3_	impregnation; treatment strategies—conditions not shown; monolithic catalyst; Vc—ascorbic acid	0.02 vol.% NH_3_, 10 vol.% O_2_, 8 vol.% CO_2_, 5 vol.% H_2_O, N_2_ balance, GHSV 100,000 h^−1^	100/28–60% (240–300 °C)	[31]
(1 wt.%)Pt/SiO_2_-Al_2_O_3_-vC			100/22–50% (240–300 °C)	
(1 wt.%)Pt/Al_2_O_3_	impregnation, calcination, 550 °C, air; monolithic catalyst	0.02 vol.% NH_3_, 8 vol.% O_2_, N_2_ balance, GHSV 100,000 h^−1^	100/15–30% (300–400 °C)	[42]
(1 wt.%)Pt/CeZrO_2_			100/20–45% (325–400 °C)	
(1.5 wt.%)Pt/ZrO_2_	impregnation, calcination, 550 °C, air	0.018 vol.% NH_3_, 8 vol.% O_2_, N_2_ balance, GHSV 100,000 h^−1^	100/25–60% (350–500 °C)	[53]
(1.5 wt.%)Pt-(5 wt.%)W/ZrO_2_			100/28–50% (300–500 °C)	
(2.0 wt.%)Pt/TiO_2_	impregnation, calcination, 400 °C, air; oxidation, 400 °C, O_2_/He	0.1 vol.% NH_3_, 4.0 vol.% O_2_, He balance, 500 mL min^−1^, mass of the catalyst: 0.145 g, WHSV 206,897 mL h^−1^ g^−1^	100/38–55% (200–400 °C)	[30]
	pulsed laser ablation in liquids, calcination, 400 °C, air oxidation, 400 °C, O_2_/He		100/22–50% (175–400 °C)	
(0.1 wt.%)Pt/(2 wt.%)V/TiO_2_	impregnation, reduction, 600 °C, H_2_/N_2_	0.02 vol.% NH_3_, 8.0 vol.% O_2_, 6.0 vol.% H_2_O, He balance, 500 mL min^−1^, mass of the catalyst: 0.25 g, GHSV 60,000 h^−1^	100/63–81% (250–350 °C)	[69]
(1.2 wt.%)Pd/Al_2_O_3_	impregnation, calcination, 600 °C, air; reduction—conditions not shown	1.14 vol.% NH_3_, 8.21 vol.% O_2_, He balance, 74.7 mL min^−1^, mass of the catalyst: 0.2 g, WHSV 22,410 mL h^−1^ g^−1^	100/98% (300 °C)	[67]
(4.2 wt.%)PdO/Al_2_O_3_	impregnation, calcination, 500 °C, air**reduction, 400 °C, H_2_/He	0.1 vol.% NH_3_, 4.0 vol.% O_2_, He balance, * 0.1 vol.% NH_3_, 4.0 vol.% O_2_, 5 vol.% H_2_O, He balance, 100 mL min^−1^, mass of the catalyst: 0.1 g, WHSV 60,000 mL h^−1^ g^−1^	100/67% (350 °C) ** 100/86% (300 °C)	[43]
(4.07 wt.%)Pd-ZSM-5	ion-exchange, calcination—conditions not shown		* 100/91% (300 °C)	
(1.5 wt.%)Pd/Y	impregnation, calcination—conditions not shown)	0.5 vol.% NH_3_, 2.5 vol.% O_2_, He balance, 40 mL min^−1^, mass of the catalyst: 0.05 g, GHSV 15,400 h^−1^	100/80–90% (250–500 °C)	[45]
(0.05 wt.%)Pt/(1 wt.%)CuO/Al_2_O_3_	impregnation, calcination air, 600 °C; impregnation, calcination air, 500 °C	0.5 vol.% NH_3_, 2.5 vol.% O_2_, He balance, 40 mL min^−1^, mass of the catalyst: 0.05 g, GHSV 15,400 h^−1^	100/44–73% (325–500 °C)	[48]
(1 wt.%)Pt/(20 wt.%)CuO/Al_2_O_3_	impregnation, calcination air, 500 °C; impregnation, calcination air, 450 °C	0.07 vol.% NH_3_, 0.5 vol.% O_2_, He balance *0.07 vol.% NH_3_, 8 vol.% O_2_, He balance 1000 mL min^−1^, WHSV 180,000 mL h^−1^ g^−1^	100/88 (210–230 °C) * 100/95 (230 °C)	[49]
(4 wt.%)Pt/(20 wt.%)CuO/Al_2_O_3_			100/83 (195–230 °C) * 100/90 (220–230 °C)	
(1.5 wt.%)Pt-(0.5 wt.%)Fe/ZSM-5	ion-exchange, calcination, air, 500 °C, impregnation calcination, air, 500 °C	0.1 vol.% NH_3_, 2 vol.% O_2_, He balance, 500 mL min^−1^, WHSV 500,000 mL h^−1^ g^−1^	100/61–88% (200–300 °C)	[55]
(0.5 wt.%)Pt-(1.54 wt.%)Fe/ZSM-5	ion-exchange, calcination, air, 500 °C, impregnation calcination, air, 500 °C	0.1 vol.% NH_3_, 2 vol.% O_2_, He balance, 500 mL min^−1^, GHSV 230,000 h^−1^	100/77–89% (250–400 °C)	[54]
(1.5 wt.%)Pt-(5.5 wt.%)Cu/ZSM-5	impregnation calcination, air, 550 °C	0.018 vol.% NH_3_, 8 vol.% O_2_, N_2_ balance, GHSV 100,000 h^−1^	100/56–73% (275–450 °C)	[53]
(0.21 wt.%)Pt/CuMgAlO*_x_*	impregnation, calcination, air, 500 °C	0.5 vol.% NH_3_, 2.5 vol.% O_2_, He balance, 40 mL min^−1^, mass of the catalyst: 0.05 g, GHSV 15,400 h^−1^	100/67–89% (350–500 °C)	[13]
(0.21 wt.%)Pd/CuMgAlO*_x_*			100/71–76% (425–500 °C)	
(0.46 wt.%)Pt/Al_2_O_3_-(0.8 wt.%)Cu-ZSM-5	Pt/Al_2_O_3_: impregnation, calcination, 500 °C, air; reduction, 500 °C, H_2_/Ar; Cu-ZSM-5: preparation not provided; oxidation, 650 °C, O_2_/Ar; monolithic catalyst *dual layer catalyst	0.05 vol.% NH_3_, 5 vol.% O_2_, Ar balance, GHSV 66,000 h^−1^	* 100/58–74% (250–500 °C)	[57]
(0.46 wt.%)Pt/Al_2_O_3_-(2.5 wt.%)Cu-ZSM-5			* 100/82–100% (250–500 °C)	
(0.46 wt.%)Pt/Al_2_O_3_-Fe-ZSM-5	Pt/Al_2_O_3_: impregnation, calcination, 500 °C, air, reduction, 500 °C, H_2_/Ar, oxidation, 650 °C, O_2_/Ar; Fe-ZSM-5: commercial; oxidation, 650 °C, O_2_/Ar; monolithic catalyst *dual layer catalyst **mixed catalyst	0.05 vol.% NH_3_, 5 vol.% O_2_, Ar balance, GHSV 66,000 h^−1^	* 100/48–93% (250–500 °C) ** 100/57–82% (250–500 °C)	[58]
(0.05 wt.%)Pt/Al_2_O_3_@(2.93 wt.%)Cu/ZSM-5	core-shell catalyst, Pt/Al_2_O_3_: impregnation, calcination, 550 °C, air; Cu-ZSM-5: ion-exchange, calcination, 500 °C, air	0.05 vol.% NH_3_, 5 vol.% O_2_, Ar balance, 100 mL min^−1^, mass of the catalyst: 0.18 g, GHSV 280,000 h^−1^	100/91–94% (310–500 °C)	[63]

## 3. Ag-Based Catalysts 

Il’chenko et al. [20,70] reported that the specific activity of metal Ag at 300 °C was lower than that of Pt and Pd. Among silver-based catalysts, γ-Al_2_O_3_ impregnated with Ag species (mainly 10 wt.%) was widely investigated. Depending on the applied conditions, i.e., catalyst (its preparation, pre-treatment strategies, etc.) and reaction conditions, the full NH_3_ conversion can be reached in the range of 150–400 °C with 45–95% N_2_ selectivity over (10 wt.%)Ag/Al_2_O_3_ (according to data gathered in Table 2). However, at temperatures above 300 °C N_2_ selectivity dropped due to the large NO production. For NH_3_-SCO, the Ag/Al_2_O_3_ catalysts are mainly applied after H_2_ pretreatment. E.g., Gang et al. [71,72] reported extremely high activity of Ag/Al_2_O_3_ at 160 °C (full NH_3_ conversion with N_2_ selectivity of about 82%), which was even superior to H_2_-reduced Ir/Al_2_O_3_ or Pt/Al_2_O_3_. The activity of Ag/Al_2_O_3_ was also higher than over silver powder and Ag/SiO_2_ [72], indicating that the applied support influenced the Ag particle dispersion. However, the difference in the Ag particle size of the Ag/Al_2_O_3_ (8.2 nm) and Ag/SiO_2_ (24 nm) catalysts was not discussed in these studies. The authors correlated the NH_3_ oxidation activity at low temperatures to the catalysts’ ability to promote dissociative or nondissociative adsorption of O_2_. However, again the role of different oxygen species (i.e., adsorbed molecular oxygen, adsorbed atomic oxygen, subsurface oxygen, and bulk dissolved oxygen) in the activity and the reaction mechanisms was not fully explored. Zhang and He [73] reported that the dissociation of O_2_ is a rate-determining step for NH_3_-SCO. They concluded that molecular O_2_ can be dissociatively chemisorbed on the surface of H_2_-reduced Ag/Al_2_O_3_, i.e., metallic species (in contrast to fresh material) to form O species, and thus enhance NH_3_-SCO activity [74]. Furthermore, the modification of Ag/Al_2_O_3_ with CeO_2_ improved catalysts’ ability in the adsorption and activation of O_2_ to form O species [75]. However, Wang et al. [76] claimed that the recovery of Brønsted acid sites via H_2_ reduction (i.e., break of Ag-O bonds on the Ag/Al_2_O_3_ surface and formation of Ag clusters in the metallic state—Ag*_n_*^0^, based on EXAFS analysis) is also responsible for the improved activity of H_2_-reduced Ag/Al_2_O_3_. Highly dispersed particles of Ag^0^ (3.5–25 nm) were found to enhance the catalytic activity below 140 °C, whereas large particles (12–50 nm) of Ag^0^ were responsible for improved N_2_ selectivity [74].

Furthermore, N_2_ selectivity of about 85% above 300 °C over (2.2 wt.%)Ag/Al_2_O_3_ (*c*(NH_3_):*c*(O_2_) = 1:1–1:25), was assigned to the small Ag particle size (<5 nm, based on XRD analysis) [77]. Ag^+^ cations are the main active species in NH_3_-SCO at temperatures above 140 °C. The adsorbed NH_3_ mainly reacts with the gaseous O_2_ over fresh Ag/Al_2_O_3_. Although besides Ag^0^ and Ag^+^, also Ag*_n_*^δ+^ species were evidenced by DR UV-Vis analysis, the authors did not specify their role in ammonia oxidation. Also, Qu et al. [78] obtained highly dispersed Ag^0^ particles with a size of 5–6 nm (based on XRD and DR UV-Vis analyses) on the H_2_-reduced (10 wt.%) Ag/Al_2_O_3_ catalyst, which reached full conversion and 89% of N_2_ selectivity at 180 °C. Yang et al. [79] and Jabłońska et al. [80] studied (1–10 wt.%)Ag/Al_2_O_3_ and claimed that the low N_2_ selectivity above 200–300 °C over Ag/Al_2_O_3_ was ascribed to the formation of Ag_2_O crystals.

Besides the influence of the Ag particle size, Wang et al. [17] investigated the effect of the different support particle size (micro-Al_2_O_3_ *versus* nano-Al_2_O_3_) on the activity of the final Ag/Al_2_O_3_ catalysts in NH_3_-SCO. The catalyst characterization indicated that nano-Al_2_O_3_ was beneficial for Ag dispersion (the average Ag particle size of 3.7 nm, based on HRTEM analysis). The catalyst abundant acid sites (based on NH_3_-TPD analysis) facilitated the adsorption and dissociation of NH_3_, therefore resulting in an enhanced activity. Furthermore, the same research group [81] studied Ag supported on TiO_2_, SiO_2_ as well as their mixture—Ag/SiO_2_-TiO_2_. Although the (10 wt.%)Ag/SiO_2_-TiO_2_ catalyst reached full NH_3_ conversion at 140 °C, N_2_ selectivity in the whole studied range of 100–240 °C was below 70%. Significantly higher N_2_ selectivity was obtained over Ag/TiO_2_ (91–99% at 180–240 °C). Jabłońska et al. [82] compared commercial TiO_2_ with mesoporous TiO_2_ (prepared by evaporation induced self-assembly (EISA)) as support in NH_3_-SCO. The activity and N_2_ selectivity were favored over (1.5 wt.%)Ag-doped mesoporous TiO_2_ (calcined at 600 °C, with predominant anatase phase). The easily reducible highly dispersed oxidized silver species were converted into Ag^0^ and possibly Ag_n_^δ+^ clusters through in situ H_2_-pretreatment of catalyst. The metallic silver decomposed N_2_O into N_2_ and surface oxygen species, leading to higher N_2_ selectivity. Further studies, concerning the stability tests, revealed that these materials are unstable, especially in the higher temperature range (>600 °C). However, temperature of the full conversion of NH_3_ over (9.8 wt.%)Ag/Al_2_O_3_ also gradually increased after ca. 4 reaction cycles (from 150 to 250 °C). A higher stability in subsequent catalytic runs revealed (9.9 wt.%)Ag/ZSM-5 with the postsynthetic modified support. The micro-/mesoporous structure could prevent sintering and/or leaching of Ag particles during NH_3_-SCO [83].

**Figure 4:**
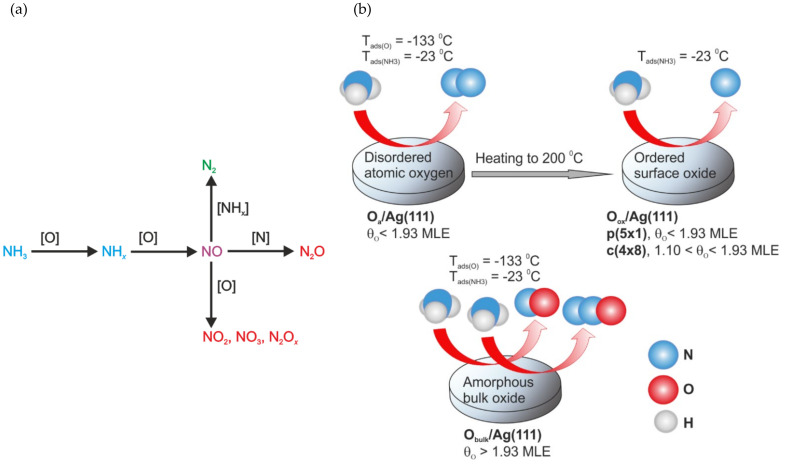
(**a**) Overall NH_3_ reaction pathway on silver powder. Reprinted from [84] with permission from Elsevier; (**b**) reactivity and selectivity trends of NH_3_-SCO on O/Ag(111) as a function of the oxygen coverage and temperature. Reprinted from [85] with permission from ACS Publications.

As stated above, while a broad number of studies examined NH_3_-SCO over Ag-based catalysts, the mechanism of NH_3_ oxidation and N_2_ formation is still uncertain, and the studies are mainly based on temperature-programmed (TPD) or in situ DRIFTS studies. E.g., Gang et al. [84] investigated NH_3_-SCO over powder silver by TPD and in situ FT-Raman spectroscopy. They found NO as the main reaction intermediate yielding N_2_O and/or N_2_ (Figure 4a). The dissociation of oxygen was believed to be the rate-controlling step for ammonia oxidation, while low surface coverage favors N_2_ formation. Similar conclusions were given by Karatok et al. [85] The exposure of ozone on Ag(111) surfaces at −133 °C led to a disordered surface atomic oxygen overlayer (confirmed by LEED). Such oxygen species selectively catalyzed N-H bond cleavage, yielding mostly N_2_ and minor amounts of by-products (NO and N_2_O). Higher coverage O/Ag(111) surfaces at −133 °C led to bulk-like amorphous silver oxide species, forming NO and N_2_O (Figure 4b). The ordered oxide surfaces—obtained through annealing of atomic oxygen-covered Ag(111) surface at 200 °C in UHV, showed only limited reactivity toward ammonia. Suppression of the N_2_ formation at high oxygen coverages was also reported over Ir(510) and Ir(110) surfaces [86].

Zhang and He [73] investigated the reaction mechanisms over Ag/Al_2_O_3_ based on in situ DRIFTS studies and found that at low temperatures (<140 °C), NH_3_ oxidation follows the -NH (imide) mechanism (Ag^0^ as the main active species), while above 140 °C, NH_3_ oxidation follows an in situ selective catalytic reduction (i-SCR) mechanism (Ag^+^ as the main active species). Furthermore, they claimed that NH_3_-SCO over Ag/nano-Al_2_O_3_ follows a reaction pathway called the N_2_^−^ mechanism (based on in situ DRIFTS, kinetic measurements, and DFT calculation results) [17]. The intermediate N_2_^−^ species appear from the combination of two -NH_2_/NH species (considered to be the rate-determining step). In the next step, the N_2_^−^ species are converted into N_2_ and/or N_2_O in the presence of O_2_.

**Figure 5:**
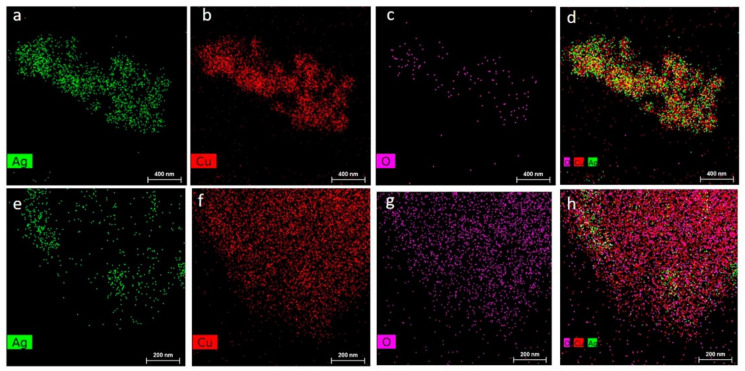
Energy-dispersive X-ray mapping for corresponding elemental distribution of Ag, Cu, and O on Ag_2_Cu_1_ (**a**–**d**) and AgCuO*_x_* NPs (**e**–**h**). Reprinted from [89] with permission from ACS Publications.

The activity and N_2_ selectivity strongly depend on the loading of noble metal and can be steered into the desired direction by the introduction of transition metal. E.g., Yang et al. [79] studied Ag-Cu/Al_2_O_3_ with 5–5 or 10–10 wt.% of metal and indicated the material with the first composition as a highly efficient catalyst (full NH_3_ conversion below 320 °C with N_2_ selectivity of more than 95%). Unfortunately, the authors did not present results of catalytic tests above 350 °C. Gang et al. [71] investigated (7.5 wt.%)Ag-(2.5 wt.%)Cu/Al_2_O_3_ and found the full conversion of ammonia at 200–300 °C with 95% N_2_ selectivity. Above 300 °C appeared significant amounts of by-products—NO and N_2_O. A mechanical mixture of (10 wt.%)Ag/Al_2_O_3_ and (10 wt.%)Cu/Al_2_O_3_—applied for comparative purposes, showed comparable activity and N_2_ selectivity to a silver-based catalyst. The same oxidation state for bimetallic (Ag-Cu/Al_2_O_3_ (5–5 wt.%, 7.5–2.5 wt.%) and monometallic (10 wt.% Ag/Al_2_O_3_ or 10 wt.% Cu/Al_2_O_3_) catalysts were approved (based on XPS analysis). Additionally, LEIS analysis over (10–2.5 wt.%, 2.5–7.5 wt.%, 5–5 wt.%, 9–1 wt.%) Ag-Cu/Al_2_O_3_ excluded formation of any Ag-Cu phases. Jabłońska et al. [80] found among all tested combinations—1–1, 1–10, 1.5–10, 5–5 wt.% of silver and copper, respectively, the (1.5 wt.%)Ag-(10 wt.%)Cu/Al_2_O_3_ catalyst with an optimum activity, N_2_ selectivity (full ammonia conversion and 94% N_2_ selectivity at 375 °C) and stability in NH_3_-SCO under wet conditions and time-on-stream tests. (0.59–2.34 wt.%)Ag-promoted CuMgAlO*_x_* hydrotalcite derived mixed metal oxides [87] with noble metal deposited inside the structure revealed relatively low NH_3_ conversion below 350 °C. Silver loading of 2.34 wt.% (*n*(Ag)/*n*(Cu)/*n*(Mg)/*n*(Al) = 1/5/65/29) led to the formation of CuO*_x_* and Ag_2_O—that caused higher catalytic activity and the observed drop in N_2_ selectivity. Significantly higher catalytic activity was reported for the Ag-Cu alloy nanoparticles (Figure 5a–d) synthesized by a solventless mix-bake-wash method. Ag-Cu (*n*(Ag)/*n*(Cu = 2/1, 77.25 wt.% of Ag) revealed full NH_3_ conversion at 200 °C. The AgCu alloy structure maintains the metallic state of Ag and Cu as well as structure stability, which enhanced activity and thermal stability in NH_3_ oxidation. The calcination of precursors of noble metal and transition metal did not form the alloy structure (Figure 5e–h). Besides, above-mentioned catalyst architectures, the (7.2 wt.%)Ag-(12.2 wt.%)Cu species were deposited onto wire-mesh honeycomb (WMH; characterized by open frontal area: 74.1%; geometric surface area: 16.2 cm^2^ cm^−3^; pressure drop: 2.58 × 10^−2^ Pa) [88]. Such catalyst revealed enhanced N_2_ selectivity (above 89% at 180–320 °C) compared to Ag/WHM as well as stability in the presence of H_2_ and CH_4_.

Concluding this part, Ag-based catalysts (especially Ag/Al_2_O_3_) received extensive concerns in NH_3_-SCO. As can be seen from the above examples, the research focuses mainly on the influence of the valance state of Ag species and particle size on the catalytic properties. As mentioned above, (10 wt.%)Ag/Al_2_O_3_ was suited for full NH_3_ conversion at about 150–400 °C with 45–95% N_2_ selectivity. Based on the presented above studies, the dispersed Ag^0^ with an average particle size in the range between 3.5–6.0 nm was found as the active species for NH_3_-SCO below 200 °C. On the other hand, there are limited studies that discuss the stability (especially concerning oxidation state) of the Ag-based catalysts, i.e., in the consecutive reaction cycles or the presence of H_2_O, SO*_x,_* and CO*_x_*. Furthermore, despite their high potential, only a few studies addressed Ag-containing bifunctional catalysts for NH_3_-SCO. A rather high content of Ag species (compared to Pt-based bifunctional catalysts, e.g., (7.5 wt.%)Ag-(2.5 wt.%)Cu/Al_2_O_3_ or (7.2 wt.%)Ag-(12.2 wt.%)Cu/WHM) was necessary to reach high activity and N_2_ selectivity (i.e., full NH_3_ conversion with 81–95% N_2_ selectivity at 200–320 °C, according to data gathered in Table 2). The catalysts containing lower content of Ag species, i.e., 0.59–1.5 wt.% required a higher temperature of 375–500 °C to fully oxidize NH_3_. Another important aspect of NH_3_-SCO over Ag-based catalysts is the investigation of the reaction mechanisms, which were explored mainly by the application of in situ DRIFTS and the indication of the characteristic intermediates of the imide, hydrazine, or i-SCR (internal) mechanism. Overall, FT-IR investigations suggest that NH_3_-SCO may follow different parallel pathways. Thus, the combination of the advantages of in situ DRIFTS with the advantages of other (e.g., temperature-programmed and/or transient) techniques will be strategic to clarify the pathways of NH_3_-SCO. Furthermore, the exploration of the reaction mechanisms should be ongoing in the realistic catalytic mixture containing besides NH_3_ and O_2_ in the inert gas also H_2_O, SO*_x,_* and CO*_x_*.

**Table 2:** Comparison of full NH_3_ conversion and N_2_ selectivity in same temperature range over Ag-based catalysts reported.

Catalyst	Catalyst Preparation	Reaction Conditions	NH_3_ Conversion_N_2_ Selectivity/% (Temperature/°C)	Ref.
Ag powder	Ag_2_O, triple reduction, 400 °C, H_2_/He; oxidation, 400 °C, O_2_/He	0.1 vol.% NH_3_, 10 vol.% O_2_, 50 mL min^−1^, mass of the catalyst: 0.1 g, WHSV 30,000 mL g^−1^ h^−1^	100/33–77% (185–400 °C)	[72]
(10 wt.%)Ag/Al_2_O_3_	impregnation, calcination, 500 °C, air; reduction, 400 °C, H_2_/He	0.1 vol.% NH_3_, 10 vol.% O_2_, 50 mL min^−1^, mass of the catalyst: 0.1 g, WHSV 30,000 mL h^−1^ g^−1^	100/70–88% (160–400 °C)	[72]
(10 wt.%)Ag/Al_2_O_3_	impregnation, calcination, 500 °C, air; *reduction, 400 °C, H_2_/N_2_	0.05 vol.% NH_3_, 10 vol.% O_2_, N_2_ balance, 100 mL min^−1^, GHSV 28,000 h^−1^	100/93–95% (180 °C) *100/80–82% (160–180 °C)	[76]
(10 wt.%)Ag/Al_2_O_3_	calcination, 600 °C, air; reduction, 400 °C, H_2_/N_2_ impregnation	0.05 vol.% NH_3_, 10 vol.% O_2_, N_2_ balance, 200 mL min^−1^, mass of the catalyst: 0.2 g, WHSV 1000 mL h^−1^ g^−1^	100/45–55% (150–200 °C)	[74]
	incipient wetness impregnation		100/55–60% (150–200 °C)	
	sol-gel		100/96% (300 °C)	
(10 wt.%)Ag/Al_2_O_3_	impregnation, calcination, 600 °C, air; reduction, 300 °C, H_2_/N_2_	0.1 vol.% NH_3_, 10 vol.% O_2_, N_2_ balance, 400 mL min^−1^, mass of the catalyst: 0.4 g, GHSV 50,000 h^−1^	100/89 (180–260 °C)	[78]
(5 wt.%)Ag/Al_2_O_3_	impregnation, calcination, 600 °C, air	0.5 vol.% NH_3_, 2.5 vol.% O_2_, Ar balance, 40 mL min^−1^, mass of the catalyst: 0.1 g, WHSV 24,000 mL h^−1^ g^−1^	100/58–83% (275–500 °C)	[80]
(10 wt.%)Ag/Al_2_O_3_	impregnation, calcination, 600 °C, air	1 vol.% NH_3_, 10 vol.% O_2_, He balance, 400 mL min^−1^, mass of the catalyst: 0.8 g, WHSV 30,000 mL h^−1^ g^−1^	100/70–83% (200–250 °C)	[79]
(10 wt.%)Ag/Al_2_O_3_	impregnation, calcination, 500 °C, air	1.14 vol.% NH_3_, 8.21 vol.% O_2_, 74.7 mL min^−1^, mass of the catalyst: 0.2 g, WHSV 22,410 mL h^−1^ g^−1^	100/83% (300 °C)	[71]
(1.5 wt.%)Ag/Al_2_O_3_	impregnation, calcination, 600 °C, air	0.5 vol.% NH_3_, 2.5 vol.% O_2_, Ar balance, 40 mL min^−1^, mass of the catalyst: 0.1 g, WHSV 24,000 mL h^−1^ g^−1^	100/78% (325 °C)	[82]
(10 wt.%)Ag/Al_2_O_3_			100/94% (225 °C)	
(2.2 wt.%)Ag/Al_2_O_3_	homogenous deposition precipitation, reduction, 400 °C, H_2_	2 vol.% NH_3_, 2 vol.% O_2_, Ar balance, 40 mL min^−1^, GHSV 2,500 h^−1^*0.15 vol.% NH_3_, 3.85 vol.% O_2_, Ar balance, 40 mL min^−1^, GHSV 2,500 h^−1^	100/100% (368–400 °C) *100/85–100% (342–400 °C)	[77]
(1.6 wt.%)Ag/CeO*_x_*/Li_2_O/Al_2_O_3_			100/95–100% (275–400 °C)	
(10 wt.%)Ag/micro-Al_2_O_3_	impregnation, calcination, 500 °C, air	0.05 vol.% NH_3_, 10 vol.% O_2_, He balance, 100 mL min^−1^, GHSV 28,000–115,000 h^−1^ *0.05 vol.% NH_3_, 10 vol.% O_2_, 5 vol.% H_2_O, He balance, 100 mL min^−1^, GHSV 136,000 h^−1^	100/94–96% (160–180 °C)	[17]
(10 wt.%)Ag/nano-Al_2_O_3_			100/66–76% (120–180 °C) *100/74–90% (250–400 °C)	
(9.8 wt.%)Ag/Al_2_O_3_	rotary evaporator, calcination, 500 °C, air; reduction, 400 °C, H_2_/Ar	0.1 vol.% NH_3_, 10 vol.% O_2_, He balance, 100 mL min^−1^, GHSV 35,000 h^−1^	100/70–74% (140–190 °C)	[83]
(9.9 wt.%)Ag/ZSM-5			100/82–89% (150–190 °C)	
(10 wt.%)Ag/SiO_2_	impregnation, calcination, 500 °C, air; reduction, 400 °C, H_2_/He	0.1 vol.% NH_3_, 10 vol.% O_2_, 50 mL min^−1^, mass of the catalyst: 0.1 g, WHSV 30,000 mL h^−1^ g^−1^	100/40–73% (200–400 °C)	[72]
(10 wt.%)Ag/SiO_2_	impregnation, calcination, 600 °C, air; reduction, 300 °C, H_2_/N_2_	0.1 vol.% NH_3_, 10 vol.% O_2_, N_2_ balance, 400 mL min^−1^, mass of the catalyst: 0.4 g, GHSV 50,000 h^−1^	100/50–63% (220–260 °C)	[78]
(10 wt.%)Ag/TiO_2_			100/64% (260 °C)	
(10 wt.%)Ag/SiO_2_	impregnation, calcination, 500 °C, air	0.05 vol.% NH_3_, 10 vol.% O_2_, N_2_ balance, 100 mL min^−1^, GHSV 28,000 h^−1^	100/60–62% (180–240 °C)	[81]
(10 wt.%)Ag/TiO_2_			100/91–99% (180–240 °C)	
(10 wt.%)Ag/SiO_2_-TiO_2_			100/60–70% (140–240 °C)	
(1.5 wt.%)Ag/mesoTiO_2_	impregnation, calcination, 600 °C, air; *reduction, 600 °C, H_2_/Ar	0.5 vol.% NH_3_, 2.5 vol.% O_2_, Ar balance, 40 mL min^−1^, mass of the catalyst: 0.1 g, WHSV 24,000 mL h^−1^ g^−1^	100/74–76% (375–400 °C) *100/81–87% (350 °C)	[82]
(10 wt.%)Ag/mesoTiO_2_			100/81–87% (350–400 °C) *100/72–78(275–400 °C)	
(7.3 wt.%)Ag/MnO_2_	multi-step process, calcination, 400 °C, air	0.005 vol.% NH_3_, 20 vol.% O_2_, Ar balance *0.005 vol.% NH_3_, 20 vol.% O_2_, 0.057 vol.% H_2_O, Ar balance 100 mL min^−1^, mass of the catalyst: 0.15 g, WHSV 40,000 mL h^−1^ g^−1^	100/98–99% (90–120 °C) *100/95–96% (115–130 °C)	[90]
(10 wt.%)Ag-Y	impregnation, calcination, 600 °C, air; reduction, 300 °C, H_2_/N_2_	0.1 vol.% NH_3_, 10 vol.% O_2_, N_2_ balance, 400 mL min^−1^, mass of the catalyst: 0.4 g, GHSV 50,000 h^−1^	100/32–50% (220–260 °C)	[78]
(21 wt.%)Ag-Y	ion-exchange, calcination, 400–500 °C, air	0.5 vol.% NH_3_, 2.5 vol.% O_2_, He balance *0.5 vol.% NH_3_, 2.5 vol.% O_2_, 3.2 vol.% H_2_O, He balance **0.5 vol.% NH_3_, 2.5 vol.% O_2_, 4.8 vol.% CO_2_, He balance 40 mL min^−1^, mass of the catalyst: 0.05 g, WHSV 48,000 mL h^−1^ g^−1^	100/90% (300 °C)	[91]
(21 wt.%)Ag-USY			100/92–95% (200–300 °C) *100/98% (200–300 °C) **100/90–95% (200–300 °C)	
(33 wt.%)Ag-Y	ion-exchange, reduction, 400 °C, H_2_	0.05 vol.% NH_3_, 7 vol.% O_2_, N_2_ balance, 800 mL min^−1^, mass of the catalyst: 0.25 g, WHSV 192,000 mL h^−1^ g^−1^	100/70–80% (300–400 °C)	[92]
(5 wt.%)Ag-(5 wt.%)Cu/Al_2_O_3_	impregnation, calcination, 600 °C, air	1 vol.% NH_3_, 10 vol.% O_2_, He balance, 400 mL min^−1^, mass of the catalyst: 0.8 g, WHSV 30,000 mL h^−1^ g^−1^	100/95% (320 °C)	[79]
(7.5 wt.%)Ag-(2.5 wt.%)Cu/Al_2_O_3_	impregnation, calcination, 500 °C, air	0.1 vol.% NH_3_, 10 vol.% O_2_, He balance, 50 mL min^−1^, mass of the catalyst: 0.1 g, WHSV 30,000 mL h^−1^ g^−1^	100/95% (200–300 °C)	[71]
(10 wt.%)Ag/Al_2_O_3_+(10 wt.%)Cu/Al_2_O_3_ (mixture 3:1)	impregnation, calcination, 500 °C, air	1.14 vol.% NH_3_, 8.21 vol.% O_2_, 74.7 mL min^−1^, mass of the catalyst: 0.2 g, WHSV 22,410 mL h^−1^ g^−1^	100/82% (300 °C)	[71]
(1.5 wt.%)Ag-(10 wt.%)Cu/Al_2_O_3_	impregnation, calcination, 600 °C, air	0.5 vol.% NH_3_, 2.5 vol.% O_2_, Ar balance *0.5 vol.% NH_3_, 2.5 vol.% O_2_, 3.2 vol.% H_2_O, Ar balance 40 mL min^−1^, mass of the catalyst: 0.1 g, WHSV 24,000 mL h^−1^ g^−1^	100/83–94% (375–500 °C) *100/83–94% (375–500 °C)	[80]
(0.59 wt.%)AgCuMgAlO*_x_*	coprecipitation, calcination, 600 °C, air	0.5 vol.% NH_3_, 2.5 vol.% O_2_, Ar balance, 40 mL min^−1^, mass of the catalyst: 0.1 g, WHSV 24,000 mL h^−1^ g^−1^	100/88% (425–500 °C)	[87]
(2.34 wt.%)AgCuMgAlO*_x_*			100/78–92% (400–500 °C)	
(77.25 wt.%)Ag-Cu nanoalloy	solventless mix-bake-wash method, 300 °C, air	0.1 vol.% NH_3_, 10 vol.% O_2_, N_2_ balance, 100 mL min^−1^, GHSV 12,000 h^−1^	100/72–85% (210–240 °C)	[89]
(67.57 wt.%)AgCuO*_x_*	calcination, 500 °C, air		100/64–72% (300–340 °C)	
(7.4 wt.%)Ag/WMH	WMH—wire-mesh honeycomb; impregnation, calcination, 500 °C, air	0.1 vol.% NH_3_, 10 vol.% O_2_, He balance, 300 mL min^−1^, GHSV 2,250 h^−1^	100/63–73% (210–320 °C)	[88]
(7.2 wt.%)Ag-(12.2 wt.%)Cu/WMH			100/81–89% (220–320 °C)	

## 4. Au- and Ru-Based Catalysts

**Figure 6:**
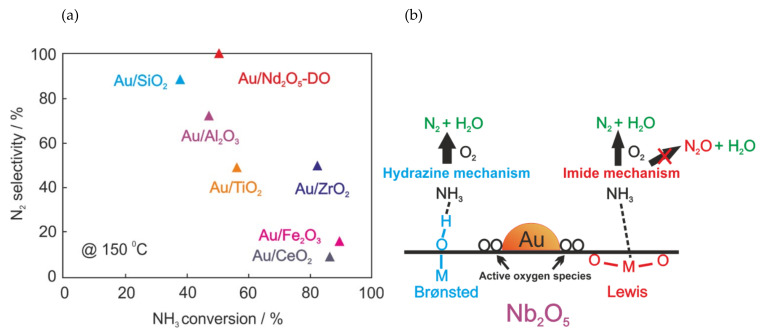
(**a**) NH_3_ conversion and N_2_ selectivity over Au/MO*_x_* at 150°C. Gold loading amount was 1 wt.%. Reaction conditions: a mass of catalyst, 0.15 g; 0.005 vol.% NH_3_ and 20 vol.% O_2_, Ar balance, GHSV 40 000 mL h^−1^ g^−1^; (**b**) Suggested NH_3_-SCO mechanisms of Au/Nb_2_O_5_. Reprinted from [12] with permission of ACS Publications.

Gold-based catalysts are well-known for their high catalytic activity at low temperatures, e.g., in the oxidation of CO [93,94]. However, such catalysts were rarely investigated in NH_3_-SCO (Table 3). Interestingly, Lin et al. [95] investigated NH_3_ oxidation over the in situ H_2_-reduced (5 wt.%)Au/MO*_x_*/Al_2_O_3_ (M = Cu, Fe, Ce, Li, and Ti) catalysts in the temperature range from 200 to 400 °C. Among all investigated catalysts, Au/Cu-Al_2_O_3_ was the most active and N_2_ selective (full NH_3_ conversion at 300 °C with 95% N_2_ selectivity). The H_2_-reduced Au/Al_2_O_3_ catalyst revealed significantly lower NH_3_ conversion (ca. 30% above 400 °C). However, the NH_3_ conversion increases with an increasing O_2_ in the feed (*c*(NH_3_):*c*(O_2_) = 1:10; NH_3_ conversion of 45% at 400 °C) [77]. Gong et al. [96] and Liu et al. [97] reported from experimental and theoretical studies that NH_3_ did not dissociate on the Au(111) surface until it was precovered with oxygen atoms or hydroxyl groups. Thus, Lippits et al. [77] observed the enhanced NH_3_ conversion (full NH_3_ conversion at 338 °C with N_2_ selectivity below 50%) over Au/Al_2_O_3_ after its doping with CeO*_x_* (able to provide and store oxygen) and Li_2_O (responsible for decreasing the catalyst acidity and thus improved oxygen adsorption). Recently, Lin et al. [12] reported the acidic metal-oxide-supported gold catalyst (Au/Nb_2_O_5_) with improved N_2_ selectivity compared to other metal-oxide-supported gold catalysts (e.g., Au/SiO_2_, Au/Al_2_O_3_, Au/Fe_2_O_3_, etc., Figure 6a). Specifically, Au/Nb_2_O_5_ contains both Brønsted and Lewis acid sites that allowed NH_3_ oxidation according to hydrazine mechanism (N_2_H_4_ as the intermediate) and imide mechanism (HNO as the intermediate), respectively (Figure 6b). Overall, further detailed property-activity studies over Au-containing materials could path the way for further catalysts design and their optimization.

**Figure 7:**
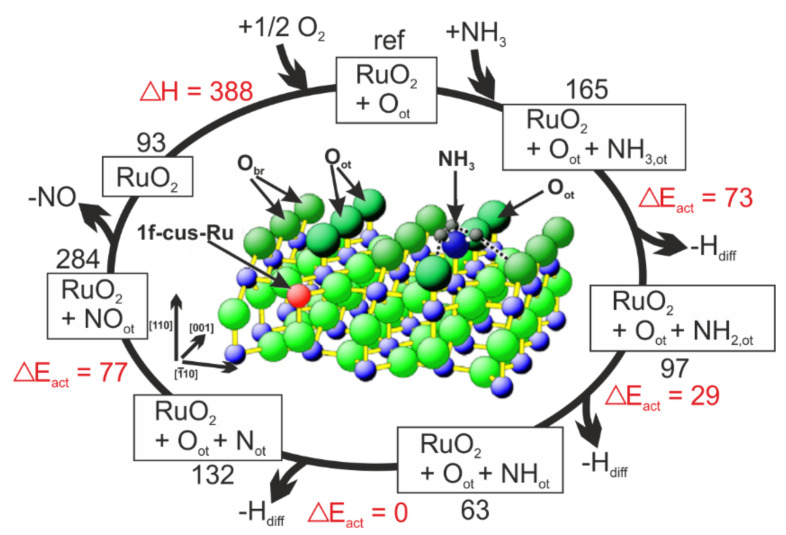
Microscopic reaction steps in NH_3_ oxidation over RuO_2_(110). Activation energies (red) and total adsorption energies (black) are determined by DFT calculations and are given in kJ mol^−1^. *-H_diff_* means that abstracted H from NH*_x_* is removed from its direct neighborhood by diffusion along with various O species on the surface. Reprinted from [99] with permission from Elsevier.

Some studies were carried out on RuO_2_(110) surface characterized by two types of atoms with unsaturated bonds along [1] direction: a) the twofold coordinated oxygen atoms (O-bridge; O-br) and b) the fivefold coordinated Ru atoms (Ru-cus; the adsorption site for ammonia) [98]. NH_3_ decomposes (to NH_2_) at −183 °C, while successive annealing to −23–27 °C produces N [98,99]. N_2_ is predominantly formed over polycrystalline RuO_2_ in a direct combination of Ru-coordinated N atoms (at ambient pressure, 6% of NO at *c*(O_2_):*c*(NH_3_) = 2:1; 65% of NO at *c*(O_2_):*c*(NH_3_) = 140:1) [100]. The selectivity to NO increases with increasing temperature (100% around 257 °C, in UHV, p(NH_3_) = 10^−7^ mbar, and *c*(O_2_):*c*(NH_3_) = 20:1) because of the high desorption temperature for NO (227 °C). At lower temperatures, NO-formation is hindered by surface water molecules [98,99]. Seitsonen et al. [99] estimated energy barriers to the elementary H-abstraction steps and the recombination of N and O atoms on RuO_2_(110) surface by using DFT calculations and high-resolution core-level shift spectroscopy (Figure 7). The high activity of RuO_2_(110) arose from low activation energies from the successive H-abstraction.

**Figure 8:**
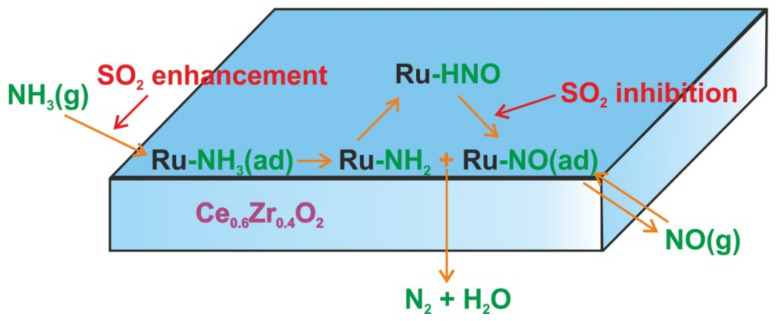
Mechanism of NH_3_-SCO and effect of SO_2_. Reprinted from [105] with permission of ACS Publications.

Also, RuO_2_-supported catalysts are active for ammonia oxidation (Table 3). E.g., Cui et al. investigated the RuO_2_-CuO/Al-ZrO_2_ [101] and CuO/RuO_2_ [102] catalysts with 5–30 wt.% and 70–95 wt.% of Ru loading, respectively. The catalysts possessed excellent activity and N_2_ selectivity at low temperatures, i.e., for (20 wt.%)RuO_2_-CuO/Al-ZrO_2_ full NH_3_ conversion at 195 °C with 100% N_2_ selectivity [101], or for (10 wt.%)CuO/RuO_2_ full NH_3_ conversion at 180 °C and N_2_ selectivity above 95% [102] were achieved. However, these catalysts are relatively expensive and therefore their commercialization is hindered. Thus, Chakrobaty et al. [103] investigated the Cu/Ru catalysts with varying overlaying thickness of Cu film (with the optimum of 0.8 monolayers) deposited by physical vapor deposition on (5 nm)Ru/TiO_2_ (111). The synergistic interaction between Cu and Ru species led to a threefold higher ammonia conversion rate than was achieved over Ru-based catalyst. Concerning the powder materials, Wang et al. [104] studied a series of WO_3_-modified RuO_2_-Fe_2_O_3_ catalysts with a lower cost, i.e., with 1 wt.% of ruthenium. The introduction of 5 wt.% of WO_3_ (among 1–9 wt.% of WO_3_) tuned the surface acidity, and thus, enhanced activity and N_2_ selectivity of RuO_2_-Fe_2_O_3_ (full NH_3_ conversion at 250–400 °C and 93–97% N_2_ selectivity). In situ DRIFTS results indicated that NH_3_-SCO over (1 wt.%)RuO_2_-(5 wt.%)WO_3_-Fe_2_O_3_ proceeds according to the i-SCR mechanism. The -NH_2_ intermediate reacted with the in situ-generated NO*_x_* ad-species with the formation of N_2_. Furthermore, Chen et al. [105,106] studied (0.2 wt.%)Ru/Ce_0.6_Zr_0.4_O_2_(PVP) or (0.2 wt.%)IrO_2_/Ce_0.6_Zr_0.4_O_2_(PVP) (PVP, polyvinylpyrrolidone) and claimed that -HNO appeared as an intermediate in the i-SCR mechanism of NH_3_-SCO (Figure 8). The formed -HNO interacted with atomic oxygen with the formation of NO, which furthermore reacts with -NH*_x_* (-NH_2_ and -NH) species with the formation of N_2_ and N_2_O (minor by-product). The presence of SO_2_ in the feed gas effectively inhibits the production of N_2_O, i.e., the reactions between gaseous NO and -NH_2_ will be enhanced (more adsorbed ammonia on the sulfated (acidic) surface). SO_2_ can also inhibit NH_3_ oxidation resulting in higher N_2_ selectivity (up to 100%) in the absence of NO*_x_*. Similar conclusions were given for RuO_x_/TiO_2_-SO_4_^2−^, however, the time of the sulfated treatment (0.5–6 h) of the support varied activity and N_2_ selectivity of the final catalysts (with an optimum at 2 h) [107].

Concluding this part, both Au- and Ru-based catalysts were significantly less investigated in NH_3_-SCO. A highly loaded Ru-containing materials (10–20 wt.% Ru; (10 wt.%)CuO-RuO_2_ or (20 wt.%)RuO_2_-CuO/Al-ZrO_2_) present a class of highly active and N_2_ selective catalysts at relatively low temperatures (i.e., full NH_3_ conversion at 180–350 °C with 95–100% N_2_ selectivity; according to data gathered in Table 3). Otherwise, the materials with significantly lower content of Ru species (0.5–3 wt.%) were less active (full NH_3_ conversion at 175–400 °C) and N_2_ selective (43–99%). However, again, concerning the influence of the preparation variables (e.g., the different total amount of ruthenium, variety of applied metal promoters and supports) as well as pretreatment and reaction conditions, the comparison of activity and N_2_ selectivity over Ru-based catalysts each other or even with other noble metal-based catalysts is limited. Furthermore, for both Au- and Ru-based catalysts, oxidized metal species (i.e., Au^+^/Au^3+^, Ru^4+^) ensure enhanced activity and N_2_ selectivity (in contrast to the Pt- or Ag-based catalysts). Nevertheless, an in-depth understanding of the role of active species in NH_3_-SCO is still lacking and needs to be demonstrated clearly in further studies (especially over Au-based catalysts). Furthermore, the presented catalytic systems were mainly investigated under ideal conditions (only NH_3_ and O_2_ diluted in inert gas) also concerning the investigation of the reaction mechanisms, i.e., through in situ DRIFTS experiments (NH_3_ adsorption/desorption in inert gas or oxygen). Otherwise, surface reactions are fast (residence time in a range of seconds) and the reaction mechanism involves a series of parallel and consecutive reactions. Thus, the reaction intermediates and conversion of the substrate molecules on the catalyst surface should be followed with more detailed ex situ, in situ, and operando spectroscopic studies as well as transient kinetic investigations under-applied reaction conditions.

**Table 3:** Comparison of full NH_3_ conversion and N_2_ selectivity in same temperature range over Au- and Ru-based catalysts reported in literature.

Catalyst	Catalyst Preparation	Reaction Conditions	NH_3_ Conversion_N_2_ Selectivity/% (Temperature/°C)	Ref.
(5 wt.%)Au/CuO/Al_2_O_3_	impregnation, calcination, 300 °C, air; reduction, 300 °C, H_2_	2 vol.% NH_3_, 2 vol.% O_2_, He balance, 30 mL min^−1^, mass of the catalyst: 0.15 g, WHSV 12,000 mL h^−1^ g^−1^	100/95% (300 °C)	[95]
(4 wt.%)Au/CeO*_x_*/Li_2_O/Al_2_O_3_	homogenous deposition precipitation, reduction, 400 °C, H_2_	2 vol.% NH_3_, 2 vol.% O_2_, Ar balance, 40 mL min^−1^, GHSV 2500 h^−1^	100/34–49% (338–400 °C)	[77]
(10 wt.%)CuO-RuO_2_	conanocasting-replication method, calcination, 500 °C, air	0.1 vol.% NH_3_, 2 vol.% O_2_, Ar balance, 100 mL min^−1^, mass of the catalyst: 0.08 g, WHSV 75,000 mL h^−1^ g^−1^	100/95–97% (180–350 °C)	[102]
(20 wt.%)RuO_2_-CuO/ZrO_2_	impregnation, calcination, 550 °C, air	0.04 vol.% NH_3_, 5 vol.% O_2_, 6 vol.% H_2_O, Ar balance, 200 mL min^−1^, mass of the catalyst: 0.1 g, WHSV 120,000 mL h^−1^ g^−1^	100/68–98% (250–275 °C)	[101]
(20 wt.%)RuO_2_/Al-ZrO_2_			100/70–82% (248–325 °C)	
(20 wt.%)RuO_2_-CuO/Al-ZrO_2_			100/100% (195–280 °C)	
(1 wt.%)RuO_2_-Fe_2_O_3_	sol-gel route, calcination, 500 °C, air	0.08 vol.% NH_3_, 5 vol.% O_2_, Ar balance, 400 mL min^−1^, GHSV 60,000 h^−1^	100/67–90% (250–400 °C)	[104]
(1 wt.%)RuO_2_-WO_3_-Fe_2_O_3_			100/93–97% (250–400 °C)	
(1.13 wt.%)Ru/Cu-SSZ-13	impregnation, 500 °C, air; pretreatment, 300 °C, O_2_/N_2_	0.05 vol.% NH_3_, 0.5 vol.% CO, 5 vol.% O_2_, N_2_ balance, GHSV 300,000 h^−1^	100/94–96% (220–300 °C)	[108]
(3 wt.%)RuO_2_/TiO_2_	impregnation, calcination, 400 °C, air	0.1 vol.% NH_3_, 5 vol.% O_2_, 3 vol.% H_2_O, N_2_ balance, 150 mL min^−1^, mass of the catalyst: 0.1 g, WHSV 90,000 mL h^−1^ g^−1^	100/47–72% (350–400 °C)	[109]
(3 wt.% Ru)RuO_2_/Na-Y	ion-exchange, pretreatment, 450 °C, O_2_/H_2_O/N_2_		100/51–99% (175–400 °C)	
(1 wt.%)Ru/TiO_2_	impregnation, 400 °C, air; pretreatment, 400 °C, O_2_/N_2_	0.02 vol.% NH_3_, 10 vol.% O_2_, 6 vol.% H_2_O, N_2_ balance, 500 mL min^−1^, mass of the catalyst: 0.3 g, GHSV 60,000 h^−1^	100/54–63% (275–300 °C)	[110]
(0.5 wt.%)Ru/TiO_2_	impregnation, 450 °C, air; pretreatment, 400 °C, O_2_/N_2_	0.08 vol.% NH_3_, 5 vol.% O_2_, N_2_ balance, 400 mL min^−1^, GHSV 60,000 h^−1^	100/43–85% (200–400 °C)	[107]
(0.5 wt.%)Ru/TiO_2_-SO_4_^2−^			100/65–93% (225–400 °C)	

## 5. Conclusions and Outlook

NH_3_-SCO is the most efficient method for ammonia removal from oxygen-containing exhausts. The number of publications related to this process successively increases with the main researchers’ interest in the development of the catalyst with high activity, N_2_ selectivity, and stability in the broad temperature range. The present mini-review provides a broad picture of the property-activity correlations of noble metal-based catalysts investigated for NH_3_-SCO. Among presented Pt-, Pd-, Ag- and Au-, Ru-based catalytic systems, mainly H_2_-reduced (1–2 wt.%)Pt/Al_2_O_3_ and (10 wt.%)Ag/Al_2_O_3_ were recognized as the most active NH_3_-SCO systems in the low temperatures (<300 °C). Unfortunately, they caused significant formation of N_2_O and NO*_x_*. Moreover, as can be seen from the above examples, although Pt- and Ag-based catalysts were more intensively investigated regarding their property-activity correlations—compared to that of the Pd-, Au-, and Ru-containing catalysts—there is still a lack of systematic studies concerning the nature and role of active species as well as the influence of (a) the particle size of active components and their aggregation state; (b) the catalyst supports (i.e., inorganic oxides versus zeolites); (c) the preparation methods (i.e., catalysts in the structured forms, e.g., monolith), and (d) feed composition (i.e., various *c*(NH_3_):*c*(O_2_) ratios (1:1–25—an excess of oxygen together with minor NH_3_ slip, presence of H_2_O, SO*_x_*, CO*_x_*, etc.) on activity, N_2_ selectivity, and stability in catalysis. Contrary to the transition metal-based catalysts only a few examples concern catalytic studies over noble metal-based zeolites. Otherwise, concerning the discussed material requirements (i.e., enhanced ammonia conversion, N_2_ selectivity and stability in the presence of typical components of exhaust gases and the broad temperature range up to 600–700 °C (in the cycle of diesel particulate filter regeneration)), the zeolite-based catalysts present a class of highly promising materials. For instance, ion-exchanged zeolites showed higher activity also in the presence of H_2_O, compared to that of alumina-supported oxides with the same metal loading due to high dispersion of metal species and acid sites of high strength. Furthermore, the studies on the reaction mechanisms are rather scarce. The reaction mechanisms must be clarified to rationally develop a process for NH_3_ oxidation to N_2_ over applied catalysts. These problems highlight the importance of more detailed ex situ and in situ methods (i.e., temperature-programmed, spectroscopic, and/or transient methods) in studying the catalysts under real working environments.

Furthermore, a relatively narrow operating temperature window of full NH_3_ conversion (Table 1, Table 2 and Table 3), high selectivity to N_2_O and NO*_x_*, and high costs of noble metals motivated researchers to develop suitable bifunctional systems. Cu-ZSM-5 or even Cu-chabazite (SSZ-13, SAPO-34) are already recognized (also discussed in the previous review articles concerning NH_3_-SCO, Jabłońska et al., 2016, Jabłońska et al., 2020) as the active and N_2_ selective catalysts for NH_3_-SCR. Despite presented here developments in bifunctional catalysts (mainly Pt-Cu or Ag-Cu catalysts systems), challenges remain in achieving enhanced NH_3_ conversion, N_2_ selectivity, and stability (in the presence of real flue gases, such as H_2_O, SO*_x_*, and CO*_x_*). Further investigations concerning bifunctional catalysts with a low number of noble metals, e.g., Au, Pt-Au, Pt-Rh, etc., constitute a promising research direction. Nevertheless, the present findings, indications, and thoughts given in the mini-review form a solid basis for further developments of structured catalysts (i.e., in the form of dual-layer or core-shell structure) and their optimization.
